# Lessons in Gynæcology

**Published:** 1887-11

**Authors:** 


### Book Reviews.

---

LESSONS IN GYNECOLOGY. By William Goodell, A. M., M. D., Professor of Clinical Gynecology in the University of Pennsylvania, etc. Third edition thoroughly revised and greatly enlarged, with one hundred and twelve illustrations. Philadelphia, Pa.: D. G. Brinton. 1887.

Goodell's Lessons in Gynecology has always been a favorite text-book with both students and practitioners of medicine. The practical character of its teaching and the lucidity of its style have won for it so wide an acquaintance that an extensive review is uncalled for. It becomes necessary, therefore, merely to note the alterations and additions which make the third edition an improvement upon previous ones.

To the chapter on "Affections of the Female Urethra" are added short sections upon Urethritis, Stricture of the Urethra and Urethrocele. The author gives a description of Emmet's "button-hole operation," but fails to recommend this procedure. In the chapter upon "Laceration of the Female Perineum" the author has modified his secondary operation for complete laceration by extending the denudation upward into the vagina along the median line, an obvious improvement. He also adds a description of Emmet's new operation, which is clearer and more satisfactory than the description given by Emmet himself. The chapter upon "Dilatation of the Cervical Canal" is greatly amplified and shows that riper and more extensive experience has fully confirmed the teaching of previous editions. We believe that Goodell's future fame will rest chiefly upon his earnest advocacy of this operation, whose value is recognized by all, except the few who follow the strange dogma recently enunciated that "obstructive dysmenorrhœa must be considered a myth."

A new chapter is added upon "Peri-uterine Inflammation." The author shares the modern skepticism in regard to the existence of pelvic cellulitis as a distinct affection and adopts the views summarized by Wm. M. Polk, that pelvic cellulitis is secondary to inflammation of the tubes and ovaries. His treatment in the first stage consists of opium, quinine, poultices and the hot vaginal douche; in the second stage, the stage of exudation of tonics internally and hot water, glycerine tampons and tincture of iodine locally. In chronic cases where solidification of the effused serum has taken place, hot vaginal douches, tincture of iodine, massage of the uterus, blisters and tonics are recommended.

A new chapter upon "Extra Ovarian Cysts" is devoted to cysts of the parovarium, cysts of the oviducts, solid tumors of the round ligament, fibroid tumor of the ovary, malignant diseases of the ovary and dermoid cyst of the ovary.

The chapters upon "Cystic Tumors of the Ovary" and "Ovariotomy" have been entirely re-written and they embody the latest and most approved ideas of the present day upon these subjects. The description of the operation of ovariotomy leaves nothing to be desired. Yet we cannot help asking if it is worth while to devote sixty-three pages to the details of an operation which can be learned only by practical observation and experience.

A new chapter upon "Hysterectomy" is added to render the work complete. As no well-established principles have been fixed for the performance of this operation, the chapter is simply a *rêsumê* of the views and methods of a few of the most prominent operators.

The additions to the work increase its size over that of the second edition by one hundred and twenty-five pages and twenty illustrations have been added. The volume is a welcome and valuable addition to American gynecological literature.

